# *Salmonella*‐infected crypt‐derived intestinal organoid culture system for host–bacterial interactions

**DOI:** 10.14814/phy2.12147

**Published:** 2014-09-11

**Authors:** Yong‐Guo Zhang, Shaoping Wu, Yinglin Xia, Jun Sun

**Affiliations:** 1 Department of Biochemistry, Rush University, 1735 W. Harrison St., Chicago, Illinois; 2 Department of Biostatistics and Computational Biology, University of Rochester, Rochester, New York; 3 Department of Internal Medicine (GI), Rush University, Chicago, Illinois; 4 Department of Microbiology/Immunology, Rush University, Chicago, Illinois

**Keywords:** Bacteria, Claudin, host–bacterial interactions, infection, inflammation, intestinal stem cells, Lgr5, NF‐*κ*B, organoid, stem cells, tight junction, ZO‐1

## Abstract

The in vitro analysis of bacterial–epithelial interactions in the intestine has been hampered by a lack of suitable intestinal epithelium culture systems. Here, we report a new experimental model using an organoid culture system to study pathophysiology of bacterial–epithelial interactions post *Salmonella* infection. Using crypt‐derived mouse intestinal organoids, we were able to visualize the invasiveness of *Salmonella* and the morphologic changes of the organoids. Importantly, we reported bacteria‐induced disruption of epithelial tight junctions in the infected organoids. In addition, we showed the inflammatory responses through activation of the NF‐*κ*B pathway in the organoids. Moreover, our western blot, PCR, and immunofluorescence data demonstrated that stem cell markers (Lgr5 and Bmi1) were significantly decreased by *Salmonella* infection (determined using GFP‐labeled Lgr5 organoids). For the first time, we created a model system that recapitulated a number of observations from in vivo studies of the *Salmonella*‐infected intestine, including bacterial invasion, altered tight junctions, inflammatory responses, and decreased stem cells. We have demonstrated that the *Salmonella*‐infected organoid culture system is a new experimental model suitable for studying host–bacterial interactions.

## Abstract (precis)

Using stem cell‐derived intestinal organoids, we were able to visualize the invasiveness of *Salmonella*, morphologic changes of the organoids, and showed the inflammatory responses through activating the NF‐*κ*B pathway in the organoids. Moreover, we show that stem cell markers (Lgr5 and Bmi1) were decreased by *Salmonella* infection. Taken together, the model system we have recreated recapitulates for the first time a number of observations from in vivo studies of *Salmonella*‐infected gut. Thus, *Salmonella*‐infected organoid culture system is a new experimental model suitable for studying host–bacterial interactions.

## Introduction

*Salmonella* cause a gastrointestinal infection known as salmonellosis. Every year, approximately 42,000 cases of salmonellosis are reported in the United States (http://www.cdc.gov/salmonella/general/). However, the in vitro analysis of *Salmonella*–host interactions still lacks suitable normal intestinal epithelium culture systems.

The intestinal epithelium is the most rapidly self‐renewing tissue in adult mammals. In the past, extensive efforts have been made to establish primary small intestinal culture systems. However, no defined, reproducible and robust culture system had been developed (Sato and Clevers 2013a,b). Most in vitro models used to investigate interactions between *Salmonella* and intestinal epithelial cells fail to recreate the differentiated tissue components and structure observed in the normal intestine. One approach to creating differentiated cells is through a suspension culture technology using a rotating wall vessel bioreactor that allows cells to remain in suspension with bubble‐free aeration. These three‐dimensional (3D) organoids are characterized by cell polarity, extracellular matrix production, and organ‐specific differentiation (Unsworth and Lelkes 1998; Hurley and McCormick 2003; Nickerson and Ott 2004; Barrila et al. 2010; Finkbeiner et al. 2012). However, this system may lack normal stem cell niches, which are responsible for the renewal of normal intestinal tissues.

Studies by Clevers and colleagues established the isolation and culture of primary small intestinal epithelial stem cells (Sato et al. 2009, 2011a,b; Sato and Clevers 2013a,b; Wang et al. 2014). In this culture system, isolated crypts form “organoid structures” with a histological hierarchy that recapitulates the in vivo small intestinal epithelium. This culture system is particularly useful for studying the regulation of intestinal stem cell self‐renewal and differentiation (Sato and Clevers 2013a,b). A recent report indicated the use of organoids as an enteric infection model for rotaviruses (Finkbeiner et al. 2012).

In the current study, we sought to establish a *Salmonella*‐infected organoid culture system using crypt‐derived intestinal organoids. We assessed the interaction of the organoids with pathogenic *Salmonella* that had previously been evaluated for pathological effects in murine models and human cell lines (Sun et al. 2004, 2005; Bruno et al. 2009; Galan 2009; Liu et al. 2010; Radtke et al. 2010; Song et al. 2010; Zhang et al. 2012). In the infected organoids, we were able to visualize the invasion of *Salmonella* and the morphologic changes of the organoids. Importantly, we reported bacteria‐induced disruption of tight junctions. We further showed the inflammatory responses through activation of the NF‐*κ*B pathway in the organoids. Moreover, our western blot, PCR, and immunofluorescence data show that stem cell marker Lgr5 was reduced by *Salmonella* infection (determined using a GFP‐labeled Lgr5 organoid system). In summary, we demonstrated that the *Salmonella*‐infected organoid culture system is a novel experimental model suitable for studying host–bacterial interactions.

## Materials and Methods

### Ethics statement

All animal work was approved by the Rush University Committee on Animal Resources. The euthanasia method used was sodium pentobarbital (100 mg per kg body weight) i.p., followed by cervical dislocation.

### Bacterial strains and growth conditions

The bacterial strains used in this study included *Salmonella enterica serovar Typhimurium* (ATCC strain 14028) and a GFP‐labeled *Salmonella* (Zhang et al. 2012). Nonagitated microaerophilic bacterial cultures were prepared as described previously (Wu et al. 2010a,b).

### Mouse intestinal organoid cell isolation, culture, and passage

The mouse small intestine (mostly jejunum and ileum) was removed immediately after cervical dislocation. The stool was then flushed out with ice‐cold PBS (penicillin, 100 I.U./mL/streptomycin, 100 *μ*g/mL), and the small intestines were dissected and opened longitudinally and cut into small (~1 cm) pieces. The tissues were rocked in PBS with 2 mmol/L EDTA for 30 min at 4°C, and then switched to PBS with 54.9 mmol/L d‐sorbitol and 43.4 mmol/L sucrose. The tissues were then vortexed for 1–2 min and filtered through a 70 *μ*m sterile cell strainer. The crypts were collected by centrifugation at 150*g* for 10 min at 4°C. Approximately 500 crypts were suspended in 50 *μ*L growth factor reduced phenol‐free Matrigel (BD Biosciences, San Jose, CA). Next, a 50 *μ*L droplet of Matrigel/crypt mix was placed in the center well of a 12‐well plate. After 30 min of polymerization, 650 *μ*L of mini gut medium was overlain (Wang et al. 2014). Mini gut medium (advanced DMEM/F12 supplemented with HEPES, l‐glutamine, N2 and B27) was added to the culture, along with R‐Spondin, Noggin, and EGF. The medium was changed every 2–3 days. For passage, organoids were removed from Matrigel and broken up mechanically by passing through a syringe and needle (27G, BD Biosciences), then transferred to fresh Matrigel. The passage was performed every 7–10 days with a 1:4 split ratio. Each experiment was repeated thrice. Each condition was examined in triplicate with multiple (>10) organoids in each sample.

### *Salmonella* colonization of organoid cells

Organoid cells (6 days after passage) were colonized with the indicated *Salmonella* strain for 30 min, washed with HBSS, and incubated in mini gut media containing gentamicin (500 mg/mL) for the indicated times, as described in our previous studies (Wu et al. 2010a,b). After extensive HBSS washing, the extracellular bacteria were washed away. Incubation with gentamicin inhibited the growth of bacteria (Sun et al. 2004). Western blot and real‐time PCR samples were collected after organoids were colonized with *Salmonella* for 30 min and then incubated in medium with gentamicin for 1 h. We found that *Salmonella* infection significantly changed the shape of organoids, including budding and the total area of the organoid cultures.

### Organoid cell immunoblotting

The organoid cells were rinsed three times in ice‐cold HBSS and then suspended in ice‐cold HBSS. The organoid cells were then spun down at 900 rpm for 10 min at 4°C. Next, using a pipette to aspirate the PBS at the top, the organoid cells were lysed in lysis buffer (1% Triton X‐100, 150 mmol/L NaCl, 10 mmol/L Tris pH 7.4, 1 mmol/L EDTA, 1 mmol/L EGTA pH 8.0, 0.2 mmol/L sodium orthovanadate, protease inhibitor cocktail) and then sonicated. The protein concentration was then measured. Next, equal amounts of protein (20 *μ*g/well) were separated by SDS‐polyacrylamide gel electrophoresis, transferred to nitrocellulose, and immunoblotted with primary antibodies. The following antibodies were used: anti‐ZO1, anti‐occludin, anti‐Claudin‐2, anti‐Claudin‐7 (Invitrogen, Carlsbad, CA), anti‐Villin, anti‐p‐P65, anti‐P65, anti‐I*κβα* (Santa Cruz, Dallas, TX), anti‐*β*‐actin (Sigma‐Aldrich, St. Louis, MO), and anti‐p‐I*κβα* (Cell Signal, Beverly, MA). Following the primary antibody step, the nitrocellulose membranes were incubated with secondary antibodies and visualized by ECL.

### Organoid cells embedded in a paraffin block

The organoid cells were rinsed three times in ice‐cold HBSS and then suspended in cold HBSS. The organoid cells were spun down at 900 rpm for 10 min at 4°C. We utilized the following protocol to fix organoid cells: 10% formalin 30 min; 75% alcohol 5 min; 100% alcohol 10 min; xylene 5 min; xylene 10 min; and paraffin (65°C) 60 min. The paraffin sections were processed with standard techniques (Ye et al. 2007; Liu et al. 2010).

### Immunofluorescence

The immunofluorescence measurements were performed on paraffin‐embedded sections (4 *μ*m) of organoid cells. After preparation of the slides, as described previously (Wu et al. 2010a,b), the slides were permeabilized for 20 min with 0.2% Triton X‐100, followed by three rinses with HBSS, and incubation for 1 h in 3% BSA + 1% goat serum in HBSS to reduce nonspecific background. The permeabilized organoid cell samples were incubated with primary antibodies overnight in a cold room. The samples were then incubated with goat anti‐rabbit Alexa Fluor 488 or goat anti‐mouse Alexa Fluor 488 (1:200; Molecular Probes, Carlsbad, CA) and DAPI (1:10,000; Molecular Probes) for 1 h at room temperature. The organoid cells were mounted with SlowFade (SlowFade^®^ AntiFade Kit, Molecular Probes) followed by a coverslip, and the edges were sealed to prevent drying. The specimens were examined with a Zeiss 710 Laser Scanning confocal microscope.

### Real‐time quantitative PCR analysis

Total RNA was extracted from organoid cells using TRIzol reagent (Invitrogen) according to the manufacturer's protocol. RNA reverse transcription (RT) was performed using the iScript cDNA synthesis kit (Bio‐Rad, Hercules, CA). The RT cDNA reaction products were subjected to quantitative real‐time PCR using the MyiQ single color real‐time PCR detection system (Bio‐Rad) and the iQ SYBR green supermix (Bio‐Rad) according to the manufacturer's protocol. The primers were used as described previously (Lu et al. 2010, 2012).

### *Salmonella*‐induced IL‐6 secretion in organoids

Organoid culture medium was obtained 1‐, 2‐, and 4‐h post *Salmonella* infection. Mouse IL‐6 was measured using the Titer‐Zyme Enzyme Immunometric Assay kit (Assay Designs, Inc., Ann Arbor, MI) according to the manufacturer's instructions.

### Fluorescence values of organoids

The GFP‐labeled Lgr5 organoids were colonized with *Salmonella* 14028s and then incubated in medium with gentamicin (500 mg/mL) for the indicated times. The organoids were suspended in cold HBSS and then sonicated with a sonifier 250 (Emerson Industrial Automation, Germany). The protein concentration was measured with a Bio‐Rad reagent concentration protein assay Dye (Bio‐Rad). Samples with equal amounts of protein were used to determine GFP fluorescence values. The fluorescence values were determined at an excitation wavelength of 395 nm and an emission wavelength of 509 nm using a Synergy H1 plate reader (BioTek, Winooski, VT). The experiments were repeated three times.

### Statistical analysis

All of the data are expressed as means ± SD. All of the statistical tests were two‐sided and *P* values of less than 0.05 were considered statistically significant. Differences between two samples were analyzed using Student's *t*‐test. For Figure 4 IL‐6, and Figure 5 fluorescence data, the two‐way ANOVA was conducted to examine the influence of treatment and time on IL‐6 secretion and fluorescence values, respectively. Both main effect and interaction effect between them were determined. After two‐way ANOVA, Tukey's test was used to adjust the *P* values for a multiple comparison. The statistical analyses were performed using SAS version 9.3 (SAS Institute, Inc., Cary, NC).

## Results

### *Salmonella* infection and invasion in mouse intestinal organoids

**Figure 1.**
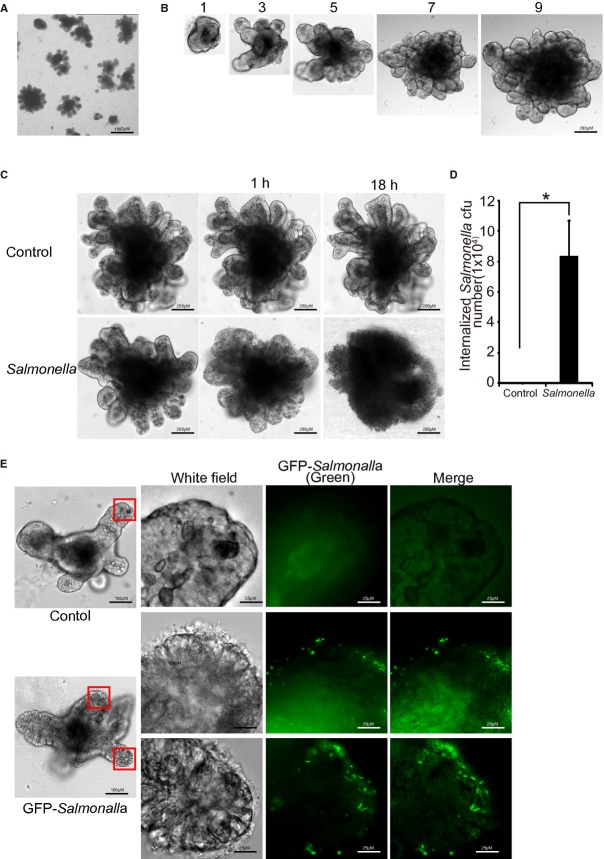
*Salmonella* infection and invasion in the mouse intestinal organoids. (A) Low magnification image of culture organoids. The organoids have been in continuous culture with regular passaging for 21 months and grow and differentiate normally. Scale bars: 1000 *μ*m, *n* = 3 repeats. Each experiment was repeated thrice. Each condition was examined in triplicate with multiple (>10) organoids in each sample. (B) Established crypt cell culture organoid model from day 1 to 9. Scale bars: 200 *μ*m. (C) The micrographs show representative organoids with or without *Salmonella* infection. Please note the morphological changes of the *Salmonella*‐infected intestinal organoids. Scale bars: 200 *μ*m, *P < 0.05, *n* = 3. Each experiment was repeated thrice. Each condition was examined in triplicate with multiple (>10) organoids in each sample. (D) Number of invasive *Salmonella* in organoids colonized with *Salmonella*. (E) *Salmonella* (green color) was able to attach and invade the culture. Scale bars: 100 *μ*m, *n* = 3. Each experiment was repeated thrice. Each condition was examined in triplicate with multiple (>10) organoids in each sample.

We established an in vitro culture of mouse small intestinal stem cells, which allows the stem cells to develop into epithelial organoids (Fig. 1A and B). We then infected the culture by colonization with pathogenic *Salmonella enterica serovar Typhimurium* 14028S (10^7^ CFU). The first 30‐min incubation allowed bacteria to contact the surface of the organoid cells. Thirty minutes later, the extracellular bacteria were washed away with Hank's balanced salt solution (HBSS). Then, the infected organoids were incubated in culture media with gentamicin for 1 h. We found that bacterial infection significantly reduced the growth of organoids, including budding and the total area of the organoid cultures (Fig. 1C). We further counted the 9.4×10^4^ CFU *Salmonella* invading after organoids colonized with *Salmonella* (Fig. 1D). To visualize the invasion of *Salmonella* in organoids, we used a GFP‐labeled *Salmonella* derived from 14028S to colonize the culture for 90 min. As shown in Figure 1E, *Salmonella* (green color) was able to attach and invade the culture.

### *Salmonella‐*induced changes of the expression of tight junction proteins

**Figure 2.**
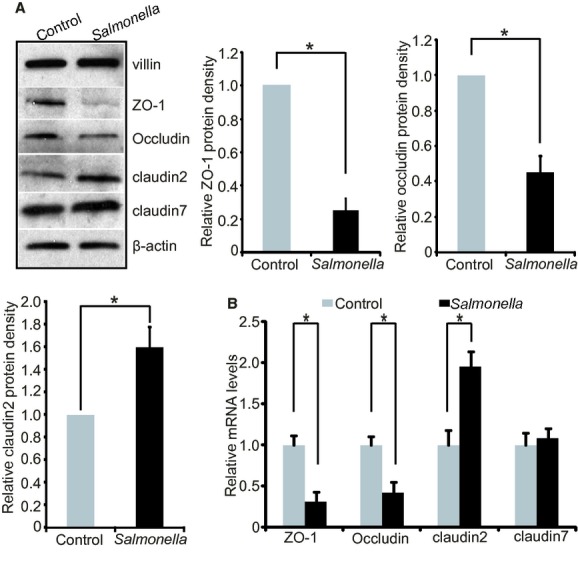
*Salmonella‐*induced changes to tight junctions. (A) Western blot analysis of tight junction protein ZO‐1, Occludin, Claudin‐2, and Claudin‐7 in infected organoids, compared to the control organoids without infection. *P < 0.05. Data are mean ± SD of the *n* = 3 repeats of control organoids or *salmonella* infection group. Each condition was examined in triplicate with multiple (>10) organoids in each sample. (B) Changes of TJ genes at the transcriptional induced by *Salmonella* infection level in the mouse intestinal organoids. At the mRNA level, ZO‐1 and Occludin were significantly decreased and Claudin‐2 was enhanced in infected organoids, whereas Claudin‐7 was not changed in the *Salmonella*‐infected organoids, as determined by quantitative PCR. Data are mean ± SD of the *n* = 3 repeats. Each experiment was repeated thrice. Each condition was examined in triplicate with multiple (>10) organoids in each sample.

*Salmonella* is known to disrupt the epithelial structure and alter tight junctions (TJ). We tested the representative TJ protein ZO‐1, Occludin, and Claudins. The results of western blot analysis indicated that the protein expression levels of ZO‐1 and Occludin were significantly decreased in infected organoids compared to the control organoids without infection (Fig. 2A). Claudin‐2 is a leaky protein that contributes to increased intestinal permeability. The protein level of Claudin‐2 was significantly increased by *Salmonella* infection, whereas Claudin‐7 was not altered in the infected organoids (Fig. 2A). At the mRNA level, ZO‐1 and Occludin were significantly decreased and Claudin‐2 was enhanced in infected organoids, whereas Claudin‐7 was not changed in the *Salmonella*‐infected organoids by quantitative PCR (Fig. 2B). The data recapitulate the observations of *Salmonella* induced TJ alteration from in vivo studies (Jepson et al. 2000; Liao et al. 2008; Zhang et al. 2013).

### Epithelial tight junctions were disrupted in the *Salmonella*‐infected intestinal organoids

**Figure 3.**
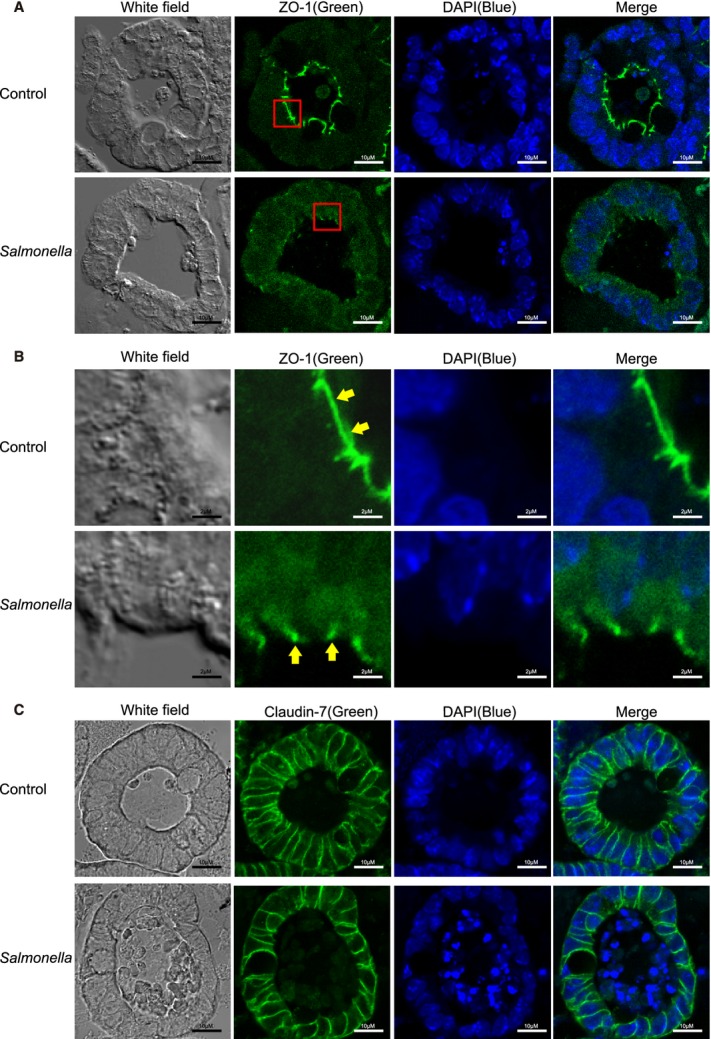
*Salmonella‐*induced disruption of tight junctions in the mouse intestinal organoids. (A) ZO‐1 distribution in organoids. Please note the disorganized structure of ZO‐1 (green staining) in organoids infected with *Salmonella*. Scale bars: 10 *μ*m. Images for ZO‐1 protein showed represent three separate experiments. (B) High magnification observation of ZO‐1. The ring‐like structure of ZO‐1 was disrupted in the mouse organoids infected by bacteria. Scale bars: 2 *μ*m. Images for ZO‐1 protein showed represent three separate experiments. (C) Distribution of Claudin‐7 was not altered by *Salmonella*. Scale bars: 10 *μ*m. Images for Claudin‐7 protein shown represent three separate experiments.

In vivo, ZO‐1 was detected at the tight junction of villous enterocytes. Our immunostaining data of ZO‐1 (Fig. 3A) showed that *Salmonella* infection disrupted the TJ structure. Yellow arrows in Figure 3B show the green staining of ZO‐1 protein on the top of the intestinal crypts of organoids. A disorganized ZO‐1 structure can be observed in the mouse organoids‐infected with *Salmonella*. Under the high magnification observed in Figure 3B, the ring‐like structure of ZO‐1 was disrupted in the mouse colon infected by bacteria. In contrast, the distribution of Claudin‐7 was not altered by *Salmonella* (Fig. 3C).

### The NF‐*κ*B pathway activation in *Salmonella*‐infected intestinal organoids

**Figure 4.**
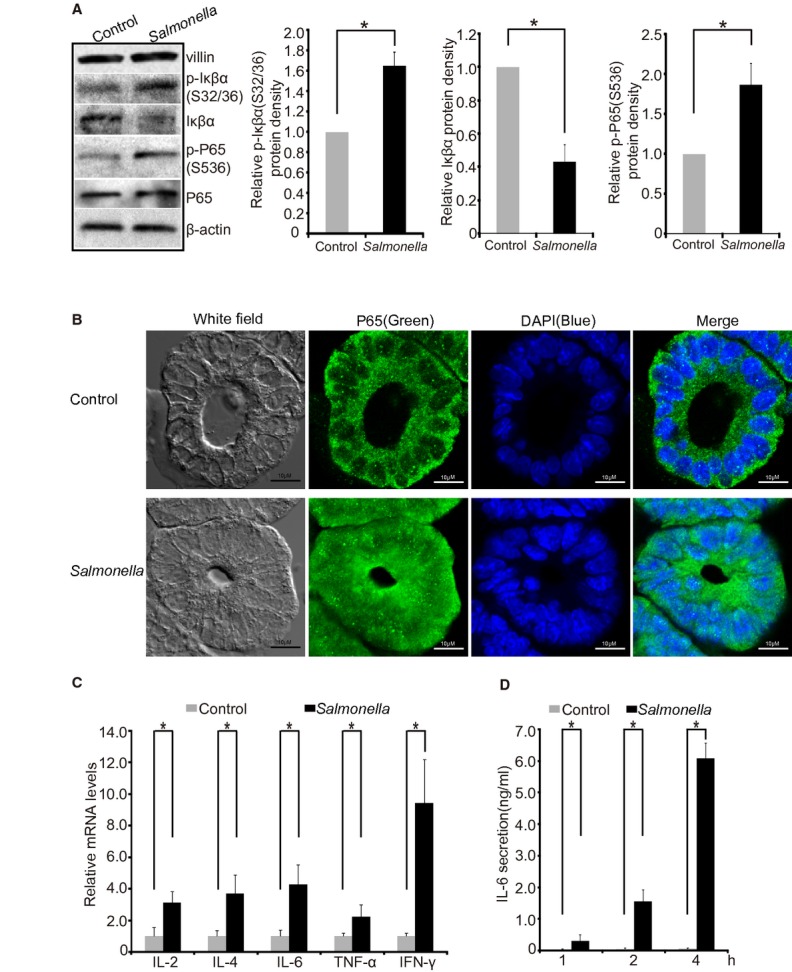
The NF‐*κ*B pathway activation in *Salmonella*‐infected intestinal organoids. (A) Western blot analysis of *Salmonella*‐infected organoids decreased the total I*κ*B*α* and increased phospho‐I*κ*B*α*. The phospho‐NF‐*κ*Bp65 was also increased, whereas total p65 was not changed in the *Salmonella*‐infected organoids. *P < 0.05. Data are mean ± SD of the *n* = 3 repeats. Each condition was examined in triplicate with multiple (>10) organoids in each sample. (B) NF‐*κ*B p65 was translocated into the nucleus in organoids infected with *Salmonella*. Scale bars: 10 *μ*m. Images for NF‐*κ*B p65 protein showed represent three separate experiments. (C) Inflammatory cytokines (IL‐2, IL‐4, IL‐6, TNF‐*α*, and IFN‐*γ*) were significantly increased in the infected organoids, compared to the organoids without any infection. (D) ELISA data of IL‐6 protein in the culture medium post 1, 2, and 4 h infection (*n* = 3 repeats). Each experiment was repeated thrice. Each condition was examined in triplicate with multiple (>10) organoids in each sample.

Nuclear factor‐*κ*B (NF‐*κ*B) is a family of transcription factors that play an essential role in innate and adaptive immune responses (Karin et al. 2002; Greten et al. 2007). NF‐*κ*B is activated in the nucleus and its activity is inhibited by the inhibitor of *κ*B*α* (I*κ*B*α*), which binds to NF‐*κ*B to block its nuclear translocation. Western blot data in Figure 4A show that *Salmonella*‐infected organoids had a significantly decreased total I*κ*B*α* and increased phospho‐I*κ*B*α*. The phospho‐NF‐*κ*B p65 was also increased, whereas total p65 was not changed in the *Salmonella*‐infected organoids. By confocal microscopy we found that NF‐κB p65 was translocated into the nucleus in organoids‐infected with *Salmonella* (Fig. 4B). As the downstream targets of NF‐κB activation, we found that inflammatory cytokines IL‐2, IL‐4, IL‐6, TNF‐*α*, and IFN‐*γ* were significantly increased in the infected organoids compared to the organoids without any infection (Fig. 4C). Moreover, ELISA data showed that the IL‐6 protein was significantly enhanced in the culture medium post 1‐, 2‐, and 4‐h infection (Fig. 4D). The ELISA is sensitive enough to detect IL‐6 protein in the culture medium 1 h post *Salmonella* infection. We did not find detectable IL‐6 production under control condition (without *Salmonella* treatment).

### *Salmonella* infection decreases stem cell marker Lgr5

**Figure 5.**
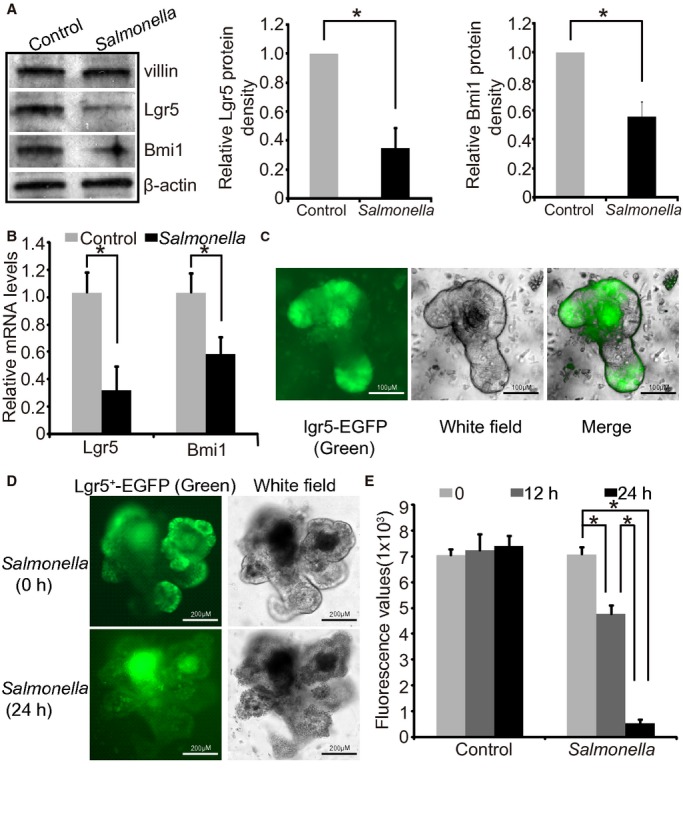
Decreased stem cell markers in *Salmonella*‐infected intestinal organoids. (A) Western blot of intestinal stem cell markers (Lgr5 and Bmi 1) in *Salmonella*‐infected intestinal organoids. *P < 0.05. Data are mean ± SD of the *n* = 3 repeats. Each experiment was repeated thrice. Each condition was examined in triplicate with multiple (>10) organoids in each sample. (B) Real‐time PCR analysis of expression of Lgr5 and Bmi 1 at the mRNA level. *P < 0.05. Data are mean ± SD of the *n* = 3 repeats. Each experiment was repeated thrice. Each condition was examined in triplicate with multiple (>10) organoids in each sample. (C) Established culture of GFP‐labeled Lgr5 organoids using GFP‐labeled Lgr5 mice. Scale bars: 100 *μ*m. Images for Lgr5‐EGFP showed represent three separate experiments. (D) Decreased immunofluorescence density of Lgr5 post *Salmonella* infection observed in the microscope. Scale bars: 200 *μ*m. Images for Lgr5‐EGFP showed represent three separate experiments. (E) *Salmonella* colonization decreased fluorescence values of organoids with GFP‐labeled Lgr5 (*n* = 3 repeats). Each experiment was repeated thrice. Each condition was examined in triplicate with multiple (>10) organoids in each sample.

*Salmonella* infection also significantly decreased the expression of intestinal stem cell marker Lgr5 and Bmi 1, as determined by PCR and western blot (Fig. 5A and B). As shown in Figure 5C, we were able to culture a GFP‐labeled Lgr5 organoids using small intestine tissue of B6.129P2‐Lgr5^tm1(cre/ERT2)Cle^/J mice (Sato et al. 2009). After *Salmonella* colonization, the fluorescence density of Lgr5 was decreased, which could be observed in the microscope (Fig. 5D). After collecting the culture, we further quantitated the fluorescence values of organoids with GFP‐labeled Lgr5. The fluorescence values decreased significantly in the *Salmonella*‐infected organoids in a time‐dependent manner (Fig. 5E). In summary, the *Salmonella*‐infected organoid culture system is a new experimental model suitable for studying host–bacterial interactions.

## Discussion

In the current study, we report the establishment of a mouse organoid model as a “mini gut” to study *Salmonella* infection and host responses to bacteria. These crypt‐derived organoids are well suited for in vitro studies of infection with *Salmonella* and provide a more physiologically relevant alternative to the two‐dimensional conventional monolayer structure. Moreover, the model system we recreated in vitro recapitulates a number of observations from in vivo studies of *Salmonella*–host interactions in the intestine (Liao et al. 2008; Lu et al. 2010; Jones and Neish 2011), including bacterial invasion, disrupted TJs, and inflammatory responses. Using organoids with or without *Salmonella* infection, we also performed analytical methods, including real‐time PCR, western blots, immunostaining, ELISA, and fluorescence values, to investigate the changes of organoids during host–bacterial interactions. The disruption of TJs, activation of inflammatory responses, and decreased stem cell marker are the same as observed in the *Salmonella*‐colitis animal model (Tam et al. 2008; Lu et al. 2010, 2012; Martinez Rodriguez et al. 2012).

Gut homeostasis is maintained through a balance between cell damage due to the collateral effects of bacterial killing and epithelial repair by stem cell division. Using a *Salmonella*‐colitis mouse model, we reported the altered location of stem cells and expression of the stem cell markers in the bacterial‐infected mammalian intestine (Liu et al. 2010; Lu et al. 2012; Martinez Rodriguez et al. 2012; Rossi et al. 2012). However, there is no reported use of an in vitro system to study bacterial infection and intestinal stem cells. Here, we reported that *Salmonella‐*infected organoids significantly decreased the expression of intestinal stem cell markers Lgr5 and Bmi1. Using GFP‐labeled Lgr5 organoids, we further visualized the change in the Lgr5‐labeled organoids and quantitated the change in fluorescence values affected by *Salmonella*. The reported system will allow us to investigate molecular mechanisms of bacterial effects on intestinal stem cells, using wild‐type and genetic engineered organoids in vitro.

In the current study, we did not test M cells in organoids. Based on a recent study (Ohno et al. 2012), RankL can induce M cell development. RankL‐induced expression of SpiB is essential for Lgr5 stem cell‐derived epithelial precursors to develop into M cells. However, RankL was not added in our organoids cultured medium. We speculate that our current organoid system perhaps is not suitable for the development of M cells. We plan to investigate M cells in our future study. We also recognize the current organoid system lacks epithelial interactions with immune cells.

To our knowledge, we report the first adaptation of the organoid model to assess the impact of *Salmonella* on crypt‐derived intestinal organoids. We chose these mouse organoids for the current studies because: (1) they mimic pathological effects observed in mouse models and human cell lines (Sato et al. 2011a,b); (2) bacterial–epithelial interactions in this model can be determined under well‐controlled experimental conditions. This organoid system can be used not only for further investigations of *Salmonella* infections but also for studies on other enteric pathogens, commensal bacteria, and probiotics; (3) this model allows us to study the bacterial infection and stem cells in vitro; and (4) the model will allow us to investigate molecular mechanisms of bacterial effects on ISCs, using wild‐type and genetic engineered organoids in vitro. Thus, in the future, we plan to develop human intestinal organoids for investigating host–bacterial interactions.

In summary, we hope that our success will spark further interest in the field for the use of the organoid model, and we expect it to provide a framework for a further understanding of how *Salmonella* infection induces intestinal inflammation. Furthermore, we anticipate that our findings related to the role of Lgr5 will assist in defining molecular mechanisms of infection and dysfunction of the stem cell niche. The *Salmonella*‐infected organoid system displays many of the cellular attributes of the corresponding in vivo tissue and, thus, provides a biologically relevant system for studying microbial pathogenesis and host responses.

## Conflict of Interest

None declared.
